# Outcomes of a community-based HIV-prevention pilot programme for township men who have sex with men in Cape Town, South Africa

**DOI:** 10.7448/IAS.16.4.18754

**Published:** 2013-12-02

**Authors:** Elizabeth Batist, Benjamin Brown, Andrew Scheibe, Stefan D Baral, Linda-Gail Bekker

**Affiliations:** 1 Desmond Tutu HIV Foundation, Cape Town, South Africa; 2 Department of Epidemiology, Johns Hopkins School of Public Health Baltimore, MD, USA

**Keywords:** community-based, self-esteem, stigma, African men who have sex with men (MSM), social network, outreach

## Abstract

**Introduction:**

Men who have sex with men (MSM) in Cape Town's townships remain in need of targeted HIV-prevention services. In 2012, a pilot community-based HIV-prevention programme was implemented that aimed to reach MSM in five Cape Town townships, disseminate HIV-prevention information and supplies, and promote the use of condoms and HIV services.

**Methods:**

Convenience sampling was used to recruit self-identified MSM who were 18 years old or older in five Cape Town townships. The six-month pilot programme trained five community leaders who, along with staff, provided HIV-prevention information and supplies to MSM through small-group meetings, community-based social activities and inter-community events. After the completion of the pilot programme, in-depth interviews and focus group discussions (FGDs) were conducted with a subset of conveniently sampled participants and with each of the community leaders. Qualitative data were then analyzed thematically.

**Results:**

Overall, 98 mostly gay-identified black MSM consented to participate, 57 community-based activities were facilitated and 9 inter-community events were conducted. Following their enrolment, 60% (59/98) of participants attended at least one pilot activity. Of those participants, 47% (28/59) attended at least half of the scheduled activities. A total of 36 participants took part in FGDs, and five in-depth interviews were completed with community leaders. Participants reported gaining access to MSM-specific HIV-prevention information, condoms and water-based lubricant through the small-group meetings. Some participants described how their feelings of loneliness, social isolation, self-esteem and self-efficacy were improved after taking part.

**Conclusions:**

The social activities and group meetings were viable strategies for disseminating HIV-prevention information, condoms and water-based lubricant to MSM in this setting. Many MSM were also able to receive social support, reduce social isolation and improve their self-esteem. Further research is needed to explore factors affecting attendance and the sustainability of these activities. Perspectives of MSM who did not attend pilot activities regularly were not equally represented in the final qualitative interviews, which could bias the findings. The use of community-based activities and small-group meetings should be explored further as components to ongoing HIV-prevention interventions for MSM in this setting.

## Introduction

Men who have sex with men (MSM) remain significantly affected by HIV in South Africa, with a reported HIV prevalence between 10 and 50% [1–6]. MSM risk is affected by many individual and structural factors, including unprotected anal intercourse (UAI), substance use and discriminatory healthcare [2–13]. Although MSM-competent HIV-prevention services are expanding across the country, there are still several gaps [1,14–17]. Reaching MSM with these services is critical for their well-being and is considered essential in addressing the broader HIV epidemic in South Africa [1,6,10,18].

Community-based approaches have been used to reach MSM and other marginalized populations with HIV-prevention services in many settings [18–21]. These programmes utilize peer education and the facilitation of safe social spaces to provide HIV education, address stigma, address behavioural risks and link individuals into HIV testing or care [18–25]. Similar strategies have been used to reach Southern African MSM with HIV research, HIV-prevention information, and HIV counselling and testing [26–32].

In South Africa, MSM-specific service providers and non-governmental organizations (NGOs), including the Desmond Tutu HIV Foundation (DTHF), engage MSM through both peer education and the use of safe spaces within township communities [13,16,30,33,34]. In 2008, the DTHF used several of these strategies to recruit MSM in Cape Town for the Global iPrEX study, a biomedical HIV-prevention clinical trial [29,35]. MSM social networks in Cape Town's townships have been described as including key individuals who establish spaces where other MSM are able to socialize safely [36]. As a result of the iPrEX study, links with these individuals and multiple MSM social networks were formed. This led the DTHF to design and conduct a pilot community-based HIV-prevention programme with MSM in these networks.

This pilot programme aimed to reach MSM in various townships through the use of community-based social activities and meeting groups. The programme was designed to disseminate HIV-prevention information and supplies, and promote the use of condoms and HIV service uptake. This article presents an overview of the project methods, a description of the participants and results from follow-up interviews and focus group discussions (FGDs) conducted with a subset of participants.

## Methods

The pilot HIV prevention programme was implemented with MSM in five predominantly black African townships in greater Cape Town. Three structured components were included: (i) group meetings were held regularly with small gatherings of MSM to facilitate knowledge exchange and disseminate prevention supplies, (ii) community-based activities were facilitated to provide opportunities for MSM group bonding and (iii) inter-community activities were conducted to promote integration and diversity. All pilot activities took place over a six-month period between May and October 2012.

### Community leader selection and participant recruitment

Townships were selected based on high HIV prevalence, which was identified through previous HIV surveillance data, and on the presence of MSM social networks identified through recruitment for the Global iPrEX study.

From each township, one MSM community leader was identified from previous research [30]. Community leaders participated in the planning and facilitation of all activities, disseminated HIV-prevention information and provided healthcare referrals to MSM in their community. They were at least 18 years old; had demonstrated leadership qualities; were respected, trusted and socially prominent among their MSM peers; and lived in a township where pilot activities were planned. The initial community leader team was selected and trained between January and April 2012.

Self-identified MSM were then recruited to take part in the pilot programme using convenience sampling through peer outreach workers and venue-based contact. All participants were 18 years old or older, were born male, were reported to have sex with men and lived in a township where the pilot was taking place. Each participant completed a self-administered paper questionnaire that collected baseline data on their demographic characteristics, sexual practices, health-seeking behaviour and access to services. Participants were offered voluntary HIV counselling and testing by trained staff and were provided with information about MSM-competent healthcare facilities. Participants who tested HIV positive were provided counselling and referrals. Participant recruitment was completed in 57 days between May and July 2012.

### Implementation of the pilot programme

Community leaders received initial two-day training and completed follow-up trainings throughout the pilot. Trainings included education on sexually transmitted infections and HIV but primarily focused on developing leadership skills such as effective communication, managing complicated social situations, strategic planning and goal setting, and encouraging healthy social norms.

Group meetings took place every 1–2 weeks and were held in private and safe venues in each township. Meetings were semi-structured and included both social and educational components such as debates about current events, training on condoms and water-based lubricant, and discussions on HIV-prevention strategies. Meetings were facilitated by a community leader and staff member but guided mostly by the participants, who were encouraged to take ownership and direction of each meeting. Condoms, water-based lubricant and HIV-prevention information were disseminated during these meetings.

Community-based activities were designed based on participant feedback and used to supplement group meetings in each township. Community-based activities included sports (hiking, netball and soccer), dance competitions, drag pageants and debates. Similar to group meetings, HIV-prevention discussions were integrated into each of the activities. Light refreshments were provided to participants at all meetings and activities.

Finally, inter-community activities, which brought together at least two different MSM groups, were conducted at least once a month. These activities were similar in scope to the community-based activities but were organized to promote knowledge sharing and socializing between MSM from different townships. MSM participants were provided with transport to attend inter-community activities.

## Data collection and analysis

### Quantitative methods

Quantitative data from the baseline questionnaires were analyzed using STATA version 11.0 (StataCorp LP, College Station, TX). Numerical variables were explored using measures of central tendency and distribution [medians and interquartile ranges (IQRs)], and categorical variables were explored using proportions and frequency tables.

Participants were requested to sign an attendance register at each activity. Registers were entered into a secure Excel spreadsheet and linked to the participant's ID. Attendance was measured for each participant and defined as the total number of events attended by the total offered to that participant.

### Qualitative methods

After completion of the pilot activities, IDIs with each of the community leaders and FGDs with a subset of participants were conducted in December 2012. A purposive sampling strategy was initially used to equally represent MSM who attended regularly and those who did not. However, many participants who did not attend regularly were unable to be contacted, resulting in the remaining FGD slots being filled by participants who attended more frequently.

All FGDs and one IDI were conducted in private facilities within each community, and four IDIs were conducted at the research offices of the DTHF. All FGDs and IDIs were conducted by one of the two trained facilitators and supported by a research assistant who took notes. The FGDs and IDIs were conducted predominantly in English, but participants were also encouraged to use the language they felt most comfortable speaking. A semi-structured interview guide was used to explore participants’ perceptions and experiences with community life, project activities, stigma, healthcare services and HIV.

Audio recordings from each FGD and IDI were transcribed, and all participant-identifying information was removed. Qualitative data were analyzed using the framework approach. Predetermined themes based on the interview guide questions were used to structure the initial framework, and a coding scheme was developed to identify emerging themes. Two analysts reviewed transcripts from one FGD and IDI together to establish consistency in coding. After this, the analysts each reviewed the remainder of the transcripts individually. Comparisons and discussion between analysts were used to reach consensus on final themes.

#### Ethical consideration

Written informed consent was obtained from all participants, who were reminded that they would be able to take part in any community-based activities regardless of their decision to participate in this pilot study. Participants taking part in the follow-up FGDs and IDIs were informed that their responses would remain anonymous and would not affect their involvement in future initiatives from the DTHF or other organizations. They received R50 (approximately US$5.00) as reimbursement for their time and transport. Community leaders were provided with a monthly stipend of R800 (approximately US$90.00) as compensation for transport costs and their time spent in project activity planning and implementation. Ethical approval for this project was obtained from the University of Cape Town's Faculty of Health Sciences Human Research Ethics Committee.

## Results

### Participant baseline characteristics

In total, 98 MSM consented to participate and completed a baseline questionnaire. The majority of participants were black African (95%, 93/98) and gay identified (82.3%, 79/96). The median age of participants was 24.5 with an IQR of 21–29. Over half of the participants had received secondary education (64.3%, 63/98), and less than one-third (28.6%, 28/98) reported current employment. High-risk sexual behaviours including UAI and transactional sex were reported by MSM in each community. In total, 26% (25/98) of participants reported having had at least one female sexual partner in the last six months. A summary of participant baseline characteristics is presented in Table 1.

**Table 1:** Participant baseline characteristics

Variable	Community A	Community B	Community C	Community D	Community E	Total
Total enrolled	33	14	24	17	10	98
Age median (IQR)	22 (19–25)	23 (20–27)	29.5 (24.5–36)	26 (24–28)	25 (21–31)	24.5 (21–29)
Race						
Black	97% (32/33)	100% (14/14)	83% (20/22)	100% (17/17)	80% (8/10)	95% (93/98)
Coloured	3% (1/33)	0%	17% (4/22)	0%	10% (1/10)	6% (6/98)
White	0%	0%	0%	0%	10% (1/10)	1% (1/98)
Sexual orientation						
Gay	78.8% (26/33)	100% (14/14)	77% (17/22)	77% (13/17)	90% (9/10)	82.3% (79/96)
Bisexual	3.0% (1/33)	0%	22% (5/22)	17.6% (3/17)	10% (1/10)	10.4% (10/96)
Straight	18.2% (6/33)	0%	0%	5.8% (1/17)	0%	7.3% (7/96)
Currently employed	24.2% (8/33)	28.6% (4/14)	20.8% (5/24)	35.2% (6/17)	50% (5/10)	28.6% (28/98)
Education						
Primary	3% (1/33)	0%	4% (1/24)	0%	10% (1/10)	3% (3/98)
Secondary	60.6% (20/33)	50% (7/14)	79% (19/24)	53% (9/17)	80% (8/10)	64.3% (63/98)
Tertiary	36.4% (12/33)	50% (7/14)	17% (4/24)	47% (8/17)	10% (1/10)	33% (32/98)
Number of male partners in last year, median (IQR)	9 (2–15)	5.5 (3–6)	6 (2.5–15)	3 (2–5)	1 (1–3)	5 (2–10)
Number of female partners in last six months, median (IQR)	2 (1–2)	1 (1–1)	2.5 (2–13)	1.5 (1–3.5)	4.5 (2–7)	2 (1–3)
Number of male partners had UAI with in the last six months, median (IQR)	2.5 (1–6)	1 (1–2)	2 (1–6)	1 (1–2)	1.5 (1–2)	2 (1–4)
Ever-reported STI	18.8% (6/32)	61.5% (8/13)	37.5% (9/25)	35.3% (6/17)	20% (2/10)	32.3% (31/96)
Transactional sex						
Paid for	24.2% (8/33)	0%	20.8% (5/24)	23.5% (4/17)	20% (2/10)	19% (19/98)
Received	24.2% (8/33)	15.4% (2/13)	33% (8/24)	19% (3/16)	20% (2/10)	24% (23/98)
Age of sexual debut, median (IQR)	15 (14–16)	17 (14–18)	17 (15–19)	17.5 (16–20)	18 (16–19)	16 (15–19)
Ever tested for HIV	90.9% (30/33)	92.8% (13/14)	100% (23/23)	94.4% (16/17)	90.0% (9/10)	93.8% (91/97)
Months since last HIV test, median (IQR)	5 (2–12)	3 (1–6)	6 (2–12)	2 (0.88–6)	7 (7–10)	4 (2–12)
Months since last visit to local clinic, median (IQR)	3 (2–6.5)	2 (1–3)	2 (1–4)	2 (0.75–5.5)	4 (.25–12)	2 (1–6)
Disclosed orientation to a healthcare worker	45.5% (15/33)	84.6% (11/13)	65.2% (15/23)	70.6% (12/17)	60% (6/10)	61.5% (59/96)
Ever visited an MSM-friendly clinic	21.2% (7/33)	100% (14/14)	70.8% (17/24)	47.1% (8/17)	30% (3/10)	50% (49/98)
Communication						
Owns a cell phone	93.9% (31/33)	92.9% (13/14)	83.3% (20/24)	100% (17/17)	90% (9/10)	91.8% (90/98)
Regular access to the internet	69.7% (23/33)	85.7% (12/14)	41.7% (10/24)	82.4% (14/17)	80% (8/10)	68.4% (67/98)
Sexual partner contact						
Via a cell phone chat programme	63.6% (21/33)	57.1% (8/14)	68% (17/25)	72.2% (13/18)	70% (7/10)	66% (66/98)
Via the internet	57.9% (19/33)	64.3% (9/14)	58.3% (14/24)	64.7% (11/17)	40% (4/10)	58% (57/98)

IQR: interquartile range; UAI: unprotected anal intercourse; STI: sexually transmitted infection; MSM: men who have sex with men.

### Community activities

MSM community groups were established in 5 townships, and 57 community-based activities including group meetings and 9 inter-community activities were conducted between May and October 2012. Participant enrolment varied between communities, with 33 participants enrolled from Community A, 24 from Community C, 17 from Community D, 14 from Community B and 10 from Community E. Less than half of the participants (44%, 43/98) had previously engaged in other MSM-focused activities or research prior to the pilot.

Attendance registers were not collected from 7 of the 57 community meetings and from one of the inter-community events due to an administrative error. A median of eight (IQR 6–9) MSM attended the 50 community meetings with attendance registers, and the eight inter-community activities were attended by a median of 20 (IQR 19.25–21.5) MSM. Condoms and lubricants were distributed during 23 activities and were available on request throughout the duration of the project. Following their enrolment, 60% (59/98) of participants attended at least one pilot activity. Of those participants, 47% (28/59) attended at least one-half of the scheduled activities. A summary of attendance is shown in Table 2.

**Table 2:** Event attendance per community

Percentage of events attended	Community 1 participants	Community 2 participants	Community 3 participants	Community 4 participants	Community 5 participants	Total
0	16	4	9	5	5	39
1–25	5	4	5	4	0	28
26–50	4	2	5	2	0	13
51–75	3	1	4	2	5	15
76–100	5	3	1	4	0	13

#### Follow-up interviews and focus group discussions

Of the 100 MSM who took part in the pilot activities, 36 also participated in follow-up FGDs, and each of the five community leaders completed an IDI. Efforts were made to include participants with varying degrees of attendance; however, there was substantial loss to follow-up of the participants who had lower attendance. Overall, more than half of the participants from the FGDs attended 50% or more of the scheduled activities.

#### HIV knowledge, testing and services

Many participants described the benefits of receiving MSM-specific HIV-prevention knowledge through the meeting groups, while others reported having already received this information elsewhere:

I didn't know everything about preventing HIV or AIDS but once I joined the group I've got more information and then that information I used it …. I was worried at first you know until I joined the group and then it influenced me in a kinda way to be strong don't have to be worried since you know that mhm theres so many things which can protect you from getting HIV. (FGD1)

Participant attitudes towards HIV testing at local health clinics remained consistently negative because of the insensitive or discriminatory care many had previously received. Despite reactions to local healthcare clinics, participants were aware and made use of MSM-competent healthcare services throughout the duration of the pilot project.

#### Use of water-based lubricants

Prior to the pilot, participants reported limited access to free water-based lubricant and described using petroleum-based lubricant during anal sex. Many participants described that their use of condoms remained inconsistent, particularly with regular sexual partners. Other participants continually referred to an improved knowledge and use of water-based lubricant as a result of taking part in the pilot activities, specifically the group meetings:

But we came to the group and they taught us that you have to use specific lube … before we can have sex. (FGD 2)

#### Social support and personal development

Participants explained how their feelings of loneliness and social isolation were improved after taking part in the pilot because it created opportunities to socialize with other MSM. This seemed particularly true in communities with little existing MSM activities.

… You are able to, you know, be yourself and the sense of getting to be yourself and also giving the feeling that you are not alone …. (FGD 1)

… It's nice when we had events, especially in our communities, because there's nothing happening so much in our community. Especially for gay people […] So the moment there is an event some people think that's where they come out to explore, “okay, I'm not the only one who's gay” …. (FGD 4)

Participants detailed how they gained meaningful social support from their peers during group meetings in each township.

When you are having this, such groups, we encourage each other, we talk to each other, we giving each other advice. So it's quite good. Whatever problem you experience in life, if you share with someone, it does help. (FGD 4)

So sometime getting together as township gay moffies, it builds us … sometimes you need to share your story with someone. That is, who will understand who you are, you understand? Because sometimes you talk to a person who's, who's a stranger in LGBTI. It doesn't work. There's no use of that because that person will look at you as you are out of your mind. (FGD 4)

Many participants shared how their self-esteem and self-efficacy improved during the meeting groups and community-based activities. For example, participants in multiple townships noted that they were able to explore and understand their sexuality, some for the first time:

I have also grown and became quite content with who I am. Cause at first, before I joined the group, I was one of those people who were in the closet as many of us would know. And […] as the time went by, I began [to be] interested in to finding out more about who I am, and why am I gay. (FGD 3)

It really did help, like for me or like, for me my family. They're religious people and they hated the fact that am gay but then I've started coming to the meetings and then I … I had the guts to tell them that now am gay …. (FGD 1)

#### Stigma

Overall, participant opinions about the role that the pilot programme played in addressing stigma were mixed, but some participants felt that the pilot activities allowed MSM to gain greater visibility in their communities.

There were outdoor events. So everybody who was even passing when we doing these kinds of activities [outdoor sporting event] were like “oh my god, this is quite interesting.” Guys playing netball, you know? […] we are trying to show the community that we are there. (FGD 3)

Participants also described how group meetings helped them to better prepare for and mitigate the effects of stigma and prejudice.

… People would like to ask question “why I'm gay?” and “why I'm doing this?” … So I'm sure this meeting helped me a lot, I mean, to get through - those kind of answers, you know what I mean? (FGD2)

If you tell your story, you talk to people then … [exhales] … it's a burden that you take off your shoulders …. (FGD 2)

#### Suggestions to improve HIV programme implementation

Participants offered suggestions for improving the implementation of the pilot activities. Specifically, they felt that staff changes should be kept minimal since it was challenging to develop relationships with new outreach staff. Some participants also expressed the need for improved efficiency with inter-community activities, specifically highlighting the transport and timeliness of other MSM as key barriers. Overall, participants also shared a willingness to engage their broader community to address stigma and expressed a need for activities to do so by targeting other community members beyond MSM.

## Discussion

This article presents the outcomes of a pilot community-based HIV-prevention programme for township MSM. It is important to note that while participants described changes in their behaviour as a result of the pilot, its aim was not to measure behaviour change. Many factors, including concurrent programmes, may have influenced participants’ behaviour [14,15]. Taking this into consideration, participant responses do suggest that this pilot was successful in achieving some of its primary objectives.

First, the pilot programme successfully engaged MSM from high-risk networks in five Cape Town townships. Attendance data suggest that social activities and group meetings were a feasible method for reaching certain MSM in this pilot; however, overall attendance varied greatly and included a large percentage of participants who attended no activities. This variability may suggest that the pilot activities did not cater to the interests or needs of all participants, particularly the unique needs of MSM [37]. Other factors that may influence attendance have been described and should be explored further in this context, including feelings of mistrust and community stigma [33,38].

Second, HIV-prevention information and supplies were successfully disseminated to MSM during this pilot. Other studies have described how facilitated social spaces can result in knowledge gain by encouraging the exchange and processing of information between peers [39]. Similarly, MSM in this pilot felt that group meetings created safe environments to learn about HIV prevention with their peers. In addition to improving knowledge, increasing access to water-based lubricants and condoms is also essential for MSM, particularly in communities where limited or incorrect lubricant use has been reported [2]. These findings support previous suggestions to explore the use of small community-based spaces for lubricant dissemination [40]. Small meeting groups and social activities should be further explored as strategies to supplement current lubricant dissemination strategies for MSM in this setting.

Third, participants reported other meaningful benefits to this pilot, including improvements in their self-efficacy, self-esteem and social isolation. Social isolation, poor self-efficacy and limited social support may play important roles in the individual risk of MSM, specifically condom negotiation and lubricant use [10,33,41,42]. Since this study did not aim to address social isolation or self-efficacy directly, it remains to be seen if any risk reduction occurred through this pilot as a result of diminished social isolation or improvements in self-efficacy. However, these results do support previous recommendations to further explore self-efficacy with township MSM in HIV-prevention programmes, and they suggest that community-based group meetings and social activities warrant further investigation as feasible methods to do so [41].

Additional research is needed to explore community-based approaches for condom use and HIV testing in this setting. HIV testing and condom use are complex behaviours affected by a multitude of factors, including stigma [10,43]. MSM in this pilot were supportive of broader community interventions to reduce stigma, lending further support to current recommendations for future community-based HIV-prevention interventions to explore methods that empower MSM to safely and appropriately address stigma within their communities [40].

There are limitations to this pilot study. This pilot targeted black African townships; therefore, these findings cannot be extrapolated to other groups. MSM who did not attend pilot activities were not equally represented in the final qualitative interviews. Their reasons for non-participation may not be adequately included in these findings. Even though participants openly shared suggestions for improving the programme, their responses may have been biased towards discussing positive benefits of the programme in general. The timeframe of this pilot was brief and cannot address the sustainability of these activities in the long term.

Taking these limitations into consideration, this community-based HIV-prevention pilot programme provides useful insights for MSM-specific HIV-prevention programming that warrant further research. Specifically, small meeting groups and social activities promoted an enabling environment, within the context of larger stigmatizing communities, where MSM were able to receive social support, improve their self-esteem and gain access to relevant HIV-prevention information and supplies.

## Conclusions

Results from this pilot programme describe how township-based MSM can benefit from facilitated social activities and meeting groups. Results from this pilot programme suggest that these strategies are a viable method for disseminating HIV-prevention information, condoms and water-based lubricant. Furthermore, these groups create a supportive environment in which MSM can learn from each other, explore their sexual identities and overcome potential barriers to HIV prevention such as social isolation and low self-esteem. The use of community-based social activities and facilitated small-group meetings should be furthered explored as components to ongoing HIV-prevention interventions for MSM in this setting.
